# Effects of short-term heat stress on the activity of three antioxidant enzymes of predatory mite *Neoseiulus barkeri* (acari, phytoseiidae)

**DOI:** 10.3389/fphys.2022.937033

**Published:** 2022-08-17

**Authors:** Wei-Zhen Li, Tong Zhu, Jing-Jiang Zhou, Su-Qin Shang

**Affiliations:** ^1^ Key Laboratory of Grassland Ecosystem of Ministry of Education, and Sino-U.S. Centers for Grazingland Ecosystem Sustainability, College of Grassland Science, Gansu Agricultural University, Lanzhou, China; ^2^ College of Plant Protection, Gansu Agricultural University/Biocontrol Engineering Laboratory of Crop Diseases and Pests of Gansu Province, Lanzhou, China; ^3^ State Key Laboratory of Green Pesticide and Agricultural Bioengineering, Ministry of Education, Guizhou University, Guiyang, China

**Keywords:** *Neoseiulus barkeri*, short-term heat stress, protein content, antioxidant enzyme, activity

## Abstract

To study the physiological mechanisms of *Neoseiulus barkeri* in response to short-term heat stress, the eggs and the emerged adults were exposed to 38, 40, and 42°C, 85% ± 5%RH,16 h:8 h (L:D) for 2, 4, and 6 h. The activities of superoxide dismutase (SOD), peroxidase (POD), and catalase (CAT) as well as the protein content of *N. barkeri* were examined. All treatments caused significant different changes compared to the untreated control. The protein content increased as the temperature increased, while it showed different changing trends with the prolongation of exposure duration. The enzymatic activity of SOD, CAT, and POD was significantly affected by the temperature treatment. Both the maximum and minimum level of the three enzymes after a short-term heat stress differed significantly to the control group (*p* < 0.05). The highest values of three enzymatic activities were all obtained at 40°C-4 h. Person correlation analysis indicates that the high temperature was the primary factor affecting the enzymatic activity, while the exposure duration of the heat stress was the secondary factor. In general, the short-term heat stress increased the protein content of *Neoseiulus barkeri* and up-regulated the expression of SOD, CAT, and POD activities as well.

## Introduction

Species of phytoseiid mites are important biological control agents against several mites and small insect pests of fruits, vegetables, and other crop species worldwide in the agriculture industry (Opit et al., 2004; Arthurs et al., 2009; Juan-Blasco et al., 2012; McMurtry et al., 2013). However, their growth and behavior are easily interfered by the ambient temperature (Hart et al., 2002; Palyvos and Emmanouel, 2009; Ghazy et al., 2012). In the context of global warming, the relationship between development rate and temperature has been used to forecast the geographical distribution of mite populations (Perring et al., 1984; Hoffmann and Sgrò, 2011). In the optimum temperature range, phytoseiid mites develop quickly, and have a high survival rate and lay more eggs (Emmert et al., 2008; Gadino and Walton, 2012). In contrary, the sudden rise of the ambient temperature will cause heat stress and exert some negative influence on the population dynamics, and lead to not only the rapid death of insects and mites, but also a decrease, even the loss, of reproduction ability (Michael and Whiting, 1996; Hoffmann et al., 2003; Cui et al., 2008).

*Neoseiulus barkeri* Hughes (Acari: Phytoseiidae), as the vital commercially produced phytoseiid mite in China, has played a key role in the control of many pest mites in recent years (Li et al., 2021). One of the deleterious consequences when *N. barkeri* are exposed to heat stress is the production of reactive oxygen species (ROS), i.e., hydroxyl radicals (HO^•−^) and superoxide anion radicals (O_2_^•−^), as well as non-radicals, such as hydrogen peroxide (H_2_O_2_) and singlet oxygen (^1^O_2_) (Yang et al., 2010; Ray et al., 2012). In normal situations, a balance exists between the generation of ROS and the antioxidant processes. However, increased levels of ROS generated inside living organisms disrupt the oxidative balance and lead to the oxidative damage. (Gechev et al., 2006; Lopez-Martinez et al., 2008). Surplus ROS, which are highly reactive and deleterious, can result in lipid peroxidation disrupting cell membrane fluidity, nucleic acids, proteins, lipids, and finally leading to apoptosis (Green and Reed, 1998; Lu et al., 2017).

To prevent damage from the ROS, mites have developed antioxidant defense mechanisms and these systems have both enzymatic and non-enzymatic components (Felton and Summers, 1995; Sorensen et al., 2001). Major components of the antioxidant enzymes of insects include superoxide dismutase (SOD) (Yucel and Kayis, 2019), catalase (CAT) (Lu et al., 2014), peroxidase (POD) (Gill and Tuteja, 2010), glutathione-S-transferases (GST) (Dubovskiy et al., 2008), polyphenol oxidase (PPO) (Tamás et al., 2017) as well as ascorbate peroxidase (APX) (Emre et al., 2013). It was reported that SOD, CAT, and POD, which are used as primary antioxidant enzymes to eliminate the ROS so as to maintain free radicals at a low level to prevent the toxic effects to living organisms (Wang et al., 2001). SOD scavenges free radicals to form H_2_O_2_ and removes the superoxide anion radical (O_2_^•−^) to prevent cells from being damaged. Whereas POD and CAT decompose H_2_O_2_, resulting in a low level of free radicals which are unable to exert a toxic effect and protecting the intact structure and function of cell membranes (Wang and Li, 2002). The change in the antioxidant enzyme activity is a significant bio indicator because of the role antioxidant enzymes play in maintaining the oxidative balance (Coskun et al., 2020).

The changes in antioxidant enzyme activity are closely associated with external stimulation and xenobiotics (e.g., pesticides, heavy metal, and high temperature) (Li et al., 2021). However, evolutionary adaptation can be rapid and may help species to counter stressful conditions or to realize ecological opportunities arising from the climate change (Bale, 2002). The evolution of insect and mite adaptability to extreme temperatures enables these organisms to withstand the effects of this environmental stress, thus supporting their survival and reproduction (Hoffmann et al., 2003).

We have found that the short-term heat stress does exert a negative effect on the longevity, reproduction characteristics, and functional response of *N. barkeri.* (Li et al., 2021; Li et al., 2022). In this study, the physiological mechanism of *N. barkeri* in the response to the short-term heat stress based on relevant antioxidant enzymes was conducted, among which the eggs and the emerged adults were treated at 38, 40, and 42°C for 2, 4, and 6 h, then the changes in the enzyme activity were tested, which aims to clarify the effects of different short-term heat stresses and exposure durations on three protective enzymes in the body of *N. barkeri*. The results would provide better understanding of revealing the physiological response mechanism of *N. barkeri* to the short-term heat stress and guidance for releasing as biological agents, and improvement in the practice of integrated control of spider mites as well.

## Materials and methods

### Mites

The initial predatory mite population of *Neoseiulus barkeri* and their prey, bran mites *Aleuroglyphus ovatus* (Acari: Sarcoptiformes), used in this study were all provided by the Institute of Fruit Tree Research in Changli, Hebei province, China. They were maintained in a climatic chamber at 25 ± 1°C, 75 ± 5% RH, L: D = 16 h: 8 h.

Newly emerged females (<24 h) were treated at 38, 40, and 42°C treated for 2, 4, and 6 h. There were 9 treatments in total (3 temperatures x 3 exposure durations). A total of six hundred survived females from each treatment were collected for the measurement of the enzyme activity. The control group was kept at room temperature (25 ± 1°C). Each treatment was replicated three times.

### The assay kits

The assay kits of superoxide (SOD), catalase (CAT), and peroxidase (POD) used in this experiment were all provided by Sino Best Biological Technology Co., Ltd, Shanghai, China.

### Preparation of the enzyme solution

The preparation of assay samples was based on the methods described by Lu et al. (2014) with some minor changes. After the short-term heat stress, 600 females of *N. barkeri* from each treatment were placed into a 2 ml centrifugal tube. A total of 1.5 ml of phosphate buffer (PBS, 0.05 M, pH 7.0) was added as an extract solution and rapidly triturated with the samples. The mixture was then centrifuged at 10,000 rpm for 15 min at 4°C. The supernatant was collected and stored at −80°C until used as the sample solution.

### Protein content determination of adults

The protein contents of sample solution were determined with the Coomassie blue method described by Bradford (1976).

The regents were sequentially added to a 96-well plate. The plate was placed in 25°C-water bath for 10 min. Then, the mixture was oscillated 120 s in a full-wavelength spectrophotometer, and the OD values of each well were read at *λ* = 595 nm (OD_595_).

### Superoxide dismutase activity assay

The regents supplied with the kit and the enzyme solution were sequentially added to a 1.5 ml glass cuvette. In the negative control, we replaced the enzyme solution with the same volume phosphate buffer (PBS, 0.05 M, pH 7.0). The cuvettes were placed in a 25°C-water bath for 30 min, and then the absorbance (OD_560_) was immediately measured and calculated using the amount of enzyme required for 50% inhibition as an enzyme activity unit (U). All the cuvettes were read at the same time. Enzyme activity was expressed as the change in the OD_560_ value per unit of time and unit of protein. This assay was replicated three times.

### Catalase activity assay

Methods were performed according to the instruction in the CAT assay kit. In a 1.5 ml glass cuvette, regents supplied by the kit and enzyme solution were sequentially added and mixed. In the negative control, we replaced the enzyme solution with the same volume phosphate buffer (PBS, 0.05 M, pH 7.0). The absorbances (A_1_) at 5 s and (A_2_) at 1 min were obtained, respectively (OD_240_). All the cuvettes were read at the same time. The degradation of 1 μmol H_2_O_2_ in 1 mg histone per minute was defined as an enzyme activity unit. This assay was replicated three times.

### Peroxidase activity assay

Methods were performed according to the instruction in the POD assay kit . The regents supplied by the kit were sequentially added to 1.5 ml glass cuvette. In the negative control, we replaced the enzyme solution with the same volume phosphate buffer (PBS, 0.05 M, pH 7.0). The instant absorbances (A_1_) at 30 s and A_2_ at 90 s were recorded (OD_470_). All the cuvettes were read at the same time. A change in absorbance at 470 nm of 0.01 per minute in 1 ml solution was defined as the enzyme activity unit. This assay was replicated three times.

### Statistical analysis

We used the online software statistical product and service Software automatically (SPSSAU 22.0, Qingsi Technology Co., LTD, Beijing, China) to detect and handle outliers before starting data analysis. The range of the box-plots were made to calculate and automatically mark the minimum and maximum estimates of the raw data. If the data exceed this range, they were regarded as outliers and deleted.

All the data were performed using SPSS software (IBM SPSS Statistics 24, IBM Corporation, Somers, NY, United States). A two-way ANOVA analysis was conducted to determine the interaction between the temperature and heat stress duration (followed by Tukey HSD post-hoc tests, *p* < 0.05). The general linear model (GML) procedure was used to analyze the means when a significant interaction was shown between the temperature and duration. In addition, the one-way ANOVA (follwed by Duncan’s new multiple range method, *p* < 0.05) was applied to test the differences among the protein content and enzyme activity. The correlation procedure in SPSS software was used to calculate the coefficients between the dependent (enzyme activity) and independent factor (temperature and exposure duration). The standard curve of bovine serum albumin calculation and figure fabrication were both conducted by GraphPad Prism 5.

## Results

The two-way ANOVA analysis indicated that the protein content was significantly affected by the temperature (*F*_(2, 81)_ = 218.303, *p* < 0.001) and the treatment duration (*F*_(2, 81)_ = 12.320, *p* = 0.032). The interaction between the temperature and duration was also significant (*F*_(4, 81)_ = 6.298, *p* < 0.001).

The protein content of *N. barkeri* females after high temperatures generally presented the variation tendency of increasing with rise of temperature are shown in Table 1. At 38°C, the protein content first increased, then decreased and then increased again, which made the value reached 0.469 mg/g from 0.165 mg/g. At 40°C, it continued to rise and reached the maximum level of 0.553 mg/g at 40°C-4 h (*F*_(1, 18)_ = 216.269, *p* < 0.001), which is more than three times higher than the control of 0.165 mg/g. Then, it descended to 0.354 mg/g but still had a significant difference with that of the control group (*F*_(1, 18)_ = 151.124, *p* = 0.002).

**TABLE 1:** Protein content of *Neoseiulus barkeri* after the short-term heat stress.

	Untreated (25°C)	38°C			40°C		
		2 h	4 h	6 h	2 h	4 h	6 h
Protein content (mg/g)	0.165 ± 0.009	0.294 ± 0.004	0.248 ± 0.011	0.469 ± 0.018	0.524 ± 0.006	0.553 ± 0.002	0.354 ± 0.013
	Ac	Bb	Be	Ab	Aa	Aa	Bc

Different capitals indicate significant differences within the different exposure duration at the same temperature, while different lowercases show significant differences within the same exposure duration at different temperature (Duncan’s new multiple range method, *p* < 0.05).

### Superoxide dismutase activity

Variance analysis showed that the SOD activity was significantly affected by temperature (*F*_(2, 81)_ = 50.257, *p* < 0.001), treatment duration (*F*_(2, 81)_ = 1.595, *p* = 0.006) as well as their interaction (*F*_(4, 81)_ = 1.228, *p* < 0.001).

**FIGURE 1:**
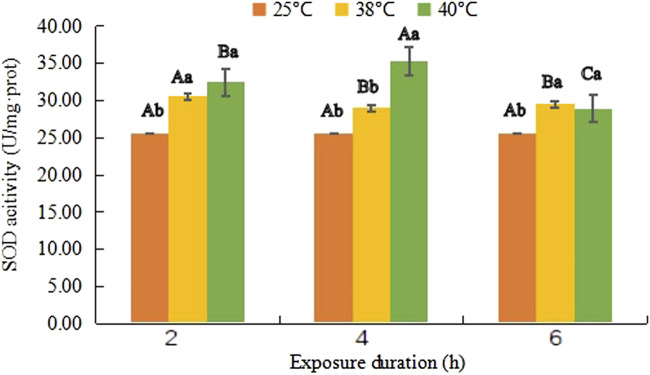
Superoxide dismutase (SOD) activity (mean activity value ± SD) in adult females of *Neoseiulus barkeri* after the short-term heat stress. Different capitals indicate significant differences within the different exposure duration at the same temperature, while different lowercases show significant differences within the same exposure duration at different temperature (Duncan’s new multiple range method, p < 0.05).

SOD activity changed differently at different temperature (Figure 1). At 38°C, it first increased to 30.55 U/mg·prot from 25.64 U/mg·prot. After slight decreases (*F*_(1, 18)_ = 20.869, *p* = 0.019) it went up again, its value reached to 29.52 U/mg·prot. However, it continued rising in the first 4 h at 40°C and reached the maximum level 35.30 U/mg·prot at 40°C- 4 h (*F*_(1, 18)_ = 56.201, *p* < 0.001), then, it dropped to 28.90 U/mg·prot but still had a significant difference from that of control (*F*_(1, 18)_ = 13.623, *p* = 0.026).

Table 2 showed the coefficients between the temperature and SOD activity, and the duration and SOD activity were 0.841 and -0.153, respectively. The correlation between the temperature and SOD activity was proved to be positive due to the positive value. On the contrary, the correlation between duration and SOD activity was negative. These results indicate that temperature was the major factor that affected the SOD activity in this experiment. No correlation was found between the two independent variables (temperature and duration), which indicated that they were mutually independent in this experiment.

**TABLE 2:** Person correlations in the SOD activity.

Variable	SOD activity	Temperature	Duration
SOD activity	1.000	0.841	−0.153
Temperature	0.841	1.000	0.000
Duration	−0.153	0.000	1.000

### Catalase activity

**FIGURE 2:**
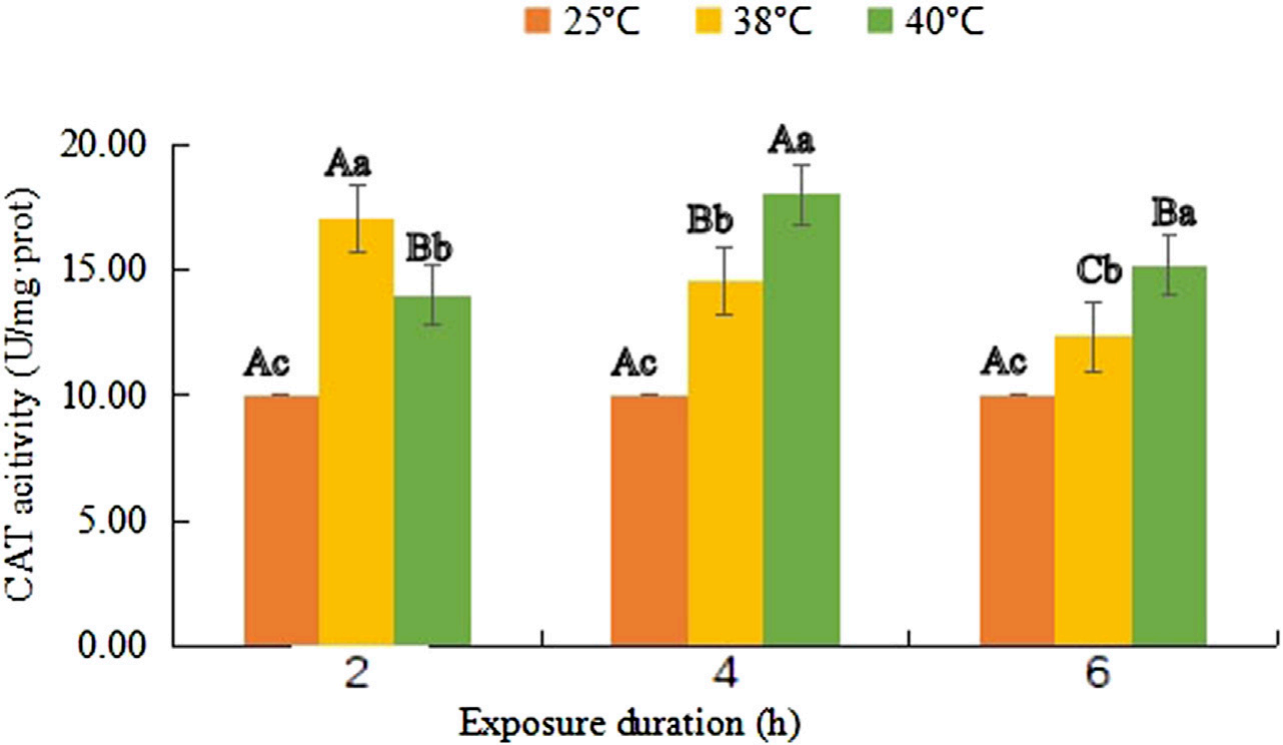
Catalase (CAT) activity (mean activity value ± SD) in adult females of *Neoseiulus barkeri* after the short-term heat stress. Different capitals indicate significant differences within the different exposure duration at the same temperature, while different lowercases show significant differences within the same exposure duration at different temperature (Duncan’s new multiple range method, p < 0.05). Variance analysis showed that the CAT activity was significantly affected by the temperature (F (2, 81) = 315.276, p < 0.001), treatment duration (F (2, 81) = 52.003, p < 0.001) as well as their interaction. (F (4, 81) = 124.198, p < 0.001).

As shown in Figure 2, the CAT enzyme activity reached a maximum value (17.03 U/mg·prot) after 2 h of treatment at 38°C, and it decreased with the prolongation of treatment time, but was still higher than that of the control group after 6 h of heat treatment (F (1, 18) = 14.747, *p* = 0.031). However, at 40°C, it increased consistently from 14.01 U/mg·prot in the first 4 h and its maximum value 18.05 U/mg·prot was obtained at 40°C-4 h (*F*_(1, 18)_ = 43.219, *p* < 0.001), and then, it dropped to 15.20 U/mg·prot (*F*_(1, 18)_ = 25.214, *p* < 0.001).

Table 3 presented the coefficients between the temperature and CAT activity, and the duration and CAT activity were 0.710 and -0.069, respectively. The temperature was also the major factor influencing the CAT activity. The exposure duration contributed less to the CAT activity variation. Similarly, no correlation was found between the temperature and duration.

**TABLE 3:** Person correlations in the CAT activity.

Variable	CAT activity	Temperature	Duration
CAT activity	1.000	0.710	−0.069
Temperature	0.710	1.000	0.000
Duration	−0.069	0.000	1.000

### Peroxidase activity

**FIGURE 3:**
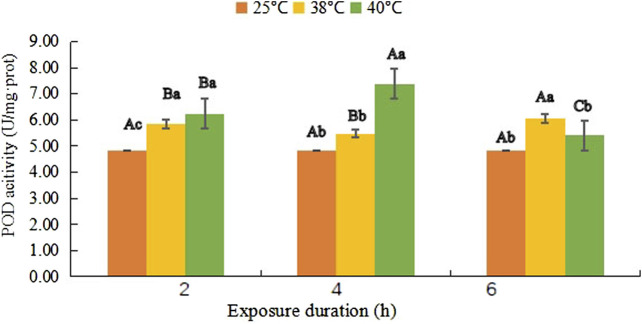
Peroxidase (POD) activity (mean activity value ± SD) in adult females of *Neoseiulus barkeri* after the short-term heat stress. Different capitals indicate significant differences within the different exposure duration at the same temperature, while different lowercases show significant differences within the same exposure duration at different temperature (Duncan’s new multiple range method, p < 0.05). Variance analysis showed that the POD activity was significantly affected by the temperature (F (2, 81) = 335.965, p < 0.001), treatment duration (F (2, 81) = 87.399, p < 0.001) as well as their interaction (F (4, 81) = 237.070, p < 0.001).

POD activity shown in Figure 3 followed the trend of increasing, then slightly decreasing (*F*_(1, 18)_ = 0.108, *p* = 0.309), and finally increasing to 6.05 U/mg·prot from 5.83 U/mg·prot, while it continued rising in the first 4 h at 40°C and reached the maximum level 7.38 U/mg·prot at 40°C- 4 h (*F*_(1, 18)_ = 62.812, *p* < 0.001), and then it dropped to 5.41 U/mg·prot, but no significant difference was found with the control of 4.83 U/mg·prot (*F*_(1, 18)_ = 1.268, *p* = 0.597).

The coefficients between the temperature and POD activity, and the duration and POD activity were shown in table 4 with values of 0.764 and −0.105, respectively, which proved that temperature was much more related than duration to the POD activity variation in this experiment. Furthermore, the temperature and duration were still completely unrelated.

**TABLE 4:** Person correlations in the POD activity.

Variable	POD activity	Temperature	Duration
POD activity	1.000	0.764	−0.105
Temperature	0.764	1.000	0.000
Duration	−0.105	0.000	1.000

## Discussion

Temperature is one of the most important environmental variables that induce physiological change in organisms (Jin et al., 2010; Jia et al., 2011). Arthropods have evolved a series of diverse behavioral and physiological strategies to avoid temperature impairments, such as seeking shelter, changing the fluidity of cell membranes, and accumulating sugars, polyols, anti-freeze proteins and amino acids (Storey, 1997; Wang and Kang, 2005; Zhang et al., 2014). *Neoseiulus barkeri*, is one of the poikilothermic organisms, whose body temperature is highly affected by the ambient temperature. Moreover, *Neoseiulus barkeri* has been playing a key role in controlling pest mites in natural filed. However, few studies of thermal stress on the *N. barkeri* were found. Our previous work and another study all showed that the thermal stress negatively affected the longevity, reproduction characteristics (Li et al., 2021), copulation (Zhang et al., 2016), and functional response (Li et al., 2022). Physiological mechanisms like the change of antioxidant enzymes of *Neoseiulus barkeri,* when faced thermal stress, were still not clear.

In the present study, the exposure of *N. barkeri* to high temperatures had significant effects on the activities of SOD, CAT, and POD. The effects were enhanced with the increasing temperature and longer exposure duration. At 38°C, the activity of three enzymes showed a sudden rise after 2 h-exposure, then the CAT continued decreasing while the SOD and POD decreased after 4 h-exposure but slightly increased after 6 h-exposure. The difference in activity between the CAT and other two enzymes could be explained that under normal physiological states, an interacting network of antioxidant enzymes effectively neutralizes the harmful effects of ROS (Davies, 1995; Kayis et al., 2015). This detoxification pathway is the result of multiple enzymes with SOD catalyzing the first step; and then, CAT, POD, and various others removing hydrogen peroxide (Sies, 1997). Specifically, SOD removes O_2_-through the process of dismutation to O_2_ and H_2_O_2_; and then, H_2_O_2_ is sequentially reduced to H_2_O and O_2_ by CAT and POD (Hoffmann et al., 1997; Kashiwagi et al., 1997).

However, the activity of the three enzymes presented the exact same variation tendency at 40°C. It consistently increased in the first 4 h and reached the maximum value, but it dropped after 6 h-exposure. It means that *N. barkeri* generated more substances like ROS; thus, enzymes with a higher level activity were needed to protect the body. Comparing with 38°C, 40°C enhanced the negative effect of thermal stress on *N. barkeri*. A similar pattern was reported by Li Z. M et al., 2010 for the activity of CAT in the adult *Tetrastichus brontispae*, a parasitoid of *Brontispa longissimi*. Significant change in POD and CAT activities were also found in the adult *Mononychellus mcgregori* after exposure to heat stress for 36–42°C (Lu et al., 2014). Thus, those activity changes of antioxidant enzymes might be the reason that *N. barkeri* could still survive at high temperature even their population characteristics were heavily affected (Coombs and Bale, 2013; Li et al., 2021).

It is known that SOD plays an important role as an antioxidant enzyme by reducing the high level of superoxide radical induced by high surrounding temperature. In this study, the SOD activity significantly increased in early exposure but decreased in the following stressing period, suggesting that SOD was induced by changes in surrounding temperatures and then protect the mites from thermal stress. In addition, *N. barkeri* might become acclimated to the thermal stress. This result is in consistent with the findings of An and Choi (2010), where early-stage of exposure to acute temperature changes resulted in the oxidative stress regulated by antioxidant enzymes, but continued stress caused by acute temperature exposure resulted in the decreased SOD activity (Yang et al., 2010). It is probably because the well-adapted characteristic that mites owned to their surrounding environment (Hangartner and Hoffmann, 2016; Xu et al., 2001).

In this study, the increased CAT and POD activity was observed together. It is believed that this increase has occurred to eliminate H_2_O_2_, which is the final product formed during the elimination of superoxide radicals via the increased SOD activity. Previous findings showed that CAT removed H_2_O_2_ only at high cellular concentrations, whereas it is inefficient for H_2_O_2_ removal at low concentrations (Ahmad et al., 1991). It is probably because the synergistic work of SOD and POD removed most of the ROS and H_2_O_2_, which made CAT remain at a low level. It has been reported that the CAT activity was too low to detect in the herbivorous citrus red mite *P. citri* when exposed to thermal stress (Yang et al., 2010). In the current study, a significant increase of CAT activities was observed in the adults of the carnivorous mite *N. barkeri* exposed to different thermal stress conditions. Apparently, it enhanced the removal of H_2_O_2_ and prevented damage by the oxidative stress. The difference in the CAT activity between the predatory mite *N. barkeri* and herbivorous mite *P. citri* suggests that the two species differ in the antioxidant defense mechanism though they both belong to the Acari (Zhang et al., 2014).

In this study, correlation analysis showed that the temperature was the primary factor affecting the enzymatic activity, while the exposure duration was the secondary factor. It is probably because the poikilotherms such as *N. barkeri* are generally well adapted to high temperatures, the longer they are exposed to a high temperature, the more adapted they would be (Hangartner and Hoffmann, 2016). The study by Feng et al. (2008) indicated that non-lethal heat pre-stimulation enhanced the ability of *Tetranychus cinnabarinus* to resist extremely high temperatures. Other similar studies have presented the same enzymatic change, but none of them pointed out which was more important between the temperature and exposure duration. These physiological changes could to some extent help the organism to resist the adverse environment, however in the meantime, they were also the processes of energy consumption at the expense of potential fertility or survival level, which might be one of the physiological explanations to their shortened longevity in our previous findings (Silbermann and Tatar, 2000; Roux et al., 2010; Zhang et al., 2013).

The present study mainly focused on the three types of primary antioxidant enzymes. Studies have shown that other substances scavenging H_2_O_2_ also exist when mites encounter heat stress, such as ascorbate peroxidase (APX) (Li et al., 2007), which has a strong affinity and binding force with H_2_O_2_, and may also be involved in the process of protecting the body. Other non-enzymes may also be used to protect the body from damage, such as ɑ -tocopherol, glutathione, trehalose, polyol, and ascorbic acid. (Ghiselli et al., 2000; Cumming et al., 2014). Antioxidant enzymes also work together with heat shock protein HSPs to reduce the ROS damage to mite bodies (Lopez-Martinez et al., 2008). These related works are expected to be conduct in the following study.

## Data Availability

The data presented in the study are deposited in the Figshare repository, accession number 10.6084/m9.figshare.19642347.
